# Expanding access to molecular oncology testing in Africa and lower middle-income countries

**DOI:** 10.4102/ajlm.v15i1.3401

**Published:** 2026-07-31

**Authors:** Sharjeel Chaudhry, Zarmina Ehtesham

**Affiliations:** 1 Dow Diagnostic Research and Reference Laboratory, Dow University of Health Sciences, Karachi, Pakistan

Cancer is a leading cause of morbidity and mortality worldwide, yet its molecular underpinnings remain insufficiently characterised in regions where the burden is rising most rapidly. Molecular oncology testing, including assays for actionable mutations, gene expression profiling, and biomarker analysis has transformed cancer care in high-income countries (HICs) by enabling precise diagnosis, prognostication, and personalised therapy. In contrast, access to these technologies in Africa and other low- and middle-income countries (LMICs) remains limited owing to systemic, infrastructural, and economic barriers, perpetuating disparities in survival and research representation.

Globally, cancer incidence in LMICs is increasing, with over 70% of new cases projected to occur in these regions by 2040.^1^ Sub-Saharan Africa alone is expected to experience a nearly 90% rise in cancer burden.^2^ Molecular biomarkers such as epidermal growth factor receptor (EGFR), anaplastic lymphoma kinase (ALK), breast cancer gene 1 and 2 (BRCA1/2), programmed death-ligand 1 (PD-L1), and Kirsten rat sarcoma viral oncogene homolog (KRAS) have redefined treatment strategies in cancers including lung, breast, and colorectal malignancies by guiding targeted therapies and immunotherapies. Without such diagnostics, treatment in LMICs remains largely empirical, limiting therapeutic efficacy.

Access barriers in Africa are multifactorial. Laboratory infrastructure for advanced molecular testing is underdeveloped, with most high-throughput sequencing platforms concentrated in a few urban centres.^3^ Fewer than 20% of national cancer centres have routine access to immunohistochemistry (IHC), and even fewer possess next-generation sequencing (NGS) capabilities.^4^ Consequently, even basic tumour subtyping remains inaccessible in many settings.

Human resource limitations compound the issue further. There is a critical shortage of trained molecular pathologists, genomic scientists, and bioinformaticians, with some regions relying on a single specialist nationwide.^5^ Training pipelines remain insufficient, and inconsistent supply chains for reagents and consumables disrupt testing continuity. Financial barriers are equally significant, as out-of-pocket healthcare expenditure dominates in many LMICs, making molecular diagnostics unaffordable for most patients.

Geographic disparities are also pronounced. Approximately 71% of NGS platforms in Africa are concentrated in five countries: South Africa, Kenya, Nigeria, Morocco, and Egypt, leaving large regions underserved.^6^ Even when available, molecular tests often impose substantial financial burdens, with patients sometimes paying more than 50% of their monthly income for essential biomarker testing.^7^ Insurance coverage for such diagnostics remains rare.

Survey data highlight these inequities further. Only 19% of LMIC facilities report adequate infrastructure for lung cancer biomarker testing, and just 27% of clinicians indicate that at least half of their patients receive molecular testing.^8^ In addition, over 80% of genomic studies are conducted in HICs, with fewer than 5% involving LMIC populations, and fewer than 3% of genome-wide association study participants being of African descent.^7,8^ This lack of representation limits the applicability of global precision oncology advances.

Despite these challenges, progress is emerging. Regional centres of excellence are developing molecular diagnostics capacity through collaborations and shared resources. Initiatives such as the Three Million African Genomes (3MAG) project aim to enhance genomic data representation and research capacity.^9^ However, these efforts remain limited in scale. Expanding access requires integration of molecular diagnostics into national cancer control programmes, supported by sustainable funding, policy frameworks, and health system strengthening. Strategies such as pooled procurement, centralised laboratory networks, and tiered diagnostic models can improve accessibility.
